# Supplementary material for: Induction of Oxidative Stress and Ferroptosis in Triple-Negative Breast Cancer Cells by Niclosamide via Blockade of the Function and Expression of SLC38A5 and SLC7A11
Source: Antioxidants (Basel). 2024 Feb 27;13(3):291. doi: 10.3390/antiox13030291 (PMC10967572; doi:10.3390/antiox13030291)
Supplement: Supplementary file 1 [file antioxidants-13-00291-s001.zip › antioxidants-2868652-supplementary.pdf]

Supplemental Material

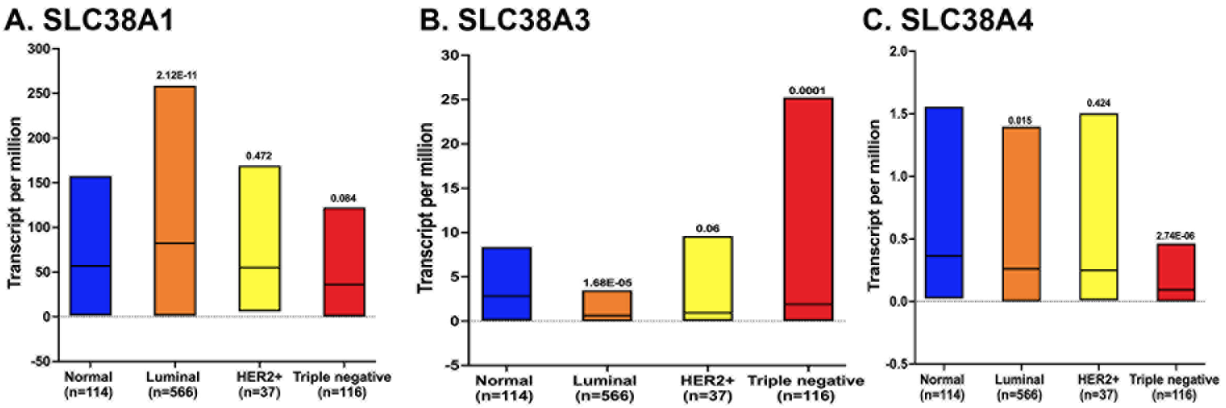

Supplemental Figure S1. Expression patterns of (**A**) SLC38A1, (**B**) SLC38A3, and (**C**) SLC38A4 mRNA in normal mammary gland and three different subtypes of breast cancer. The data were culled from the TCGA database. The numbers in parentheses represent the number of cases in each category. The p value is given on top of each bar for the respective breast cancer subtype compared to normal mammary gland.

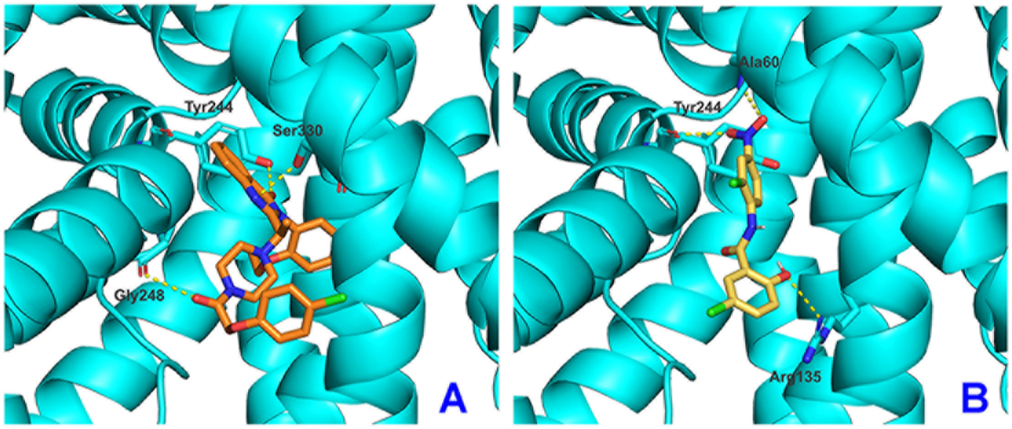

Supplemental Figure S2. For structural modeling of the cystine/glutamate antiporter, we have used the recently reported cryo-EM crystal structure of human transporter-chaperone complex SLC7A11/SLC3A2 (PDB: 7P9V). Docking simulations were conducted using AutoDock/Vina in conjunction with the USCF Chimera program. (**A**) Docking of erastin with SLC7A11. (**B**) Docking of niclosamide with SLC7A11. The amino acid residues in SLC7A11 that interact with the ligands are indicated for each ligand.

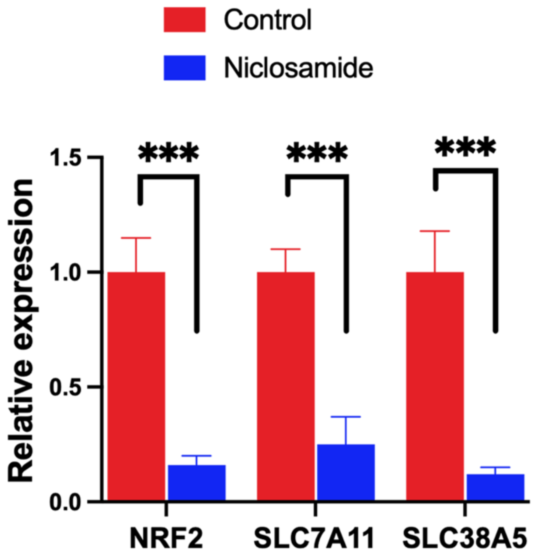

Supplemental Figure S3. Relative expression levels of mRNAs for Nrf2, SLC7A11, and SLC38A5 in control tumors and niclosamide-exposed tumors. MB231 cells were used for xenografts and niclosamide was administered to mice in the experimental group at a dose of 4 mg/kg by daily intraperitoneal injection. At the end of the experimental period described in Fig. 11, RNA was prepared from the tumor tissues and used for qRT-PCR. 18S mRNA was used as the internal control. Data are expressed as mean ± S.E. ***, $p < 0.001$.

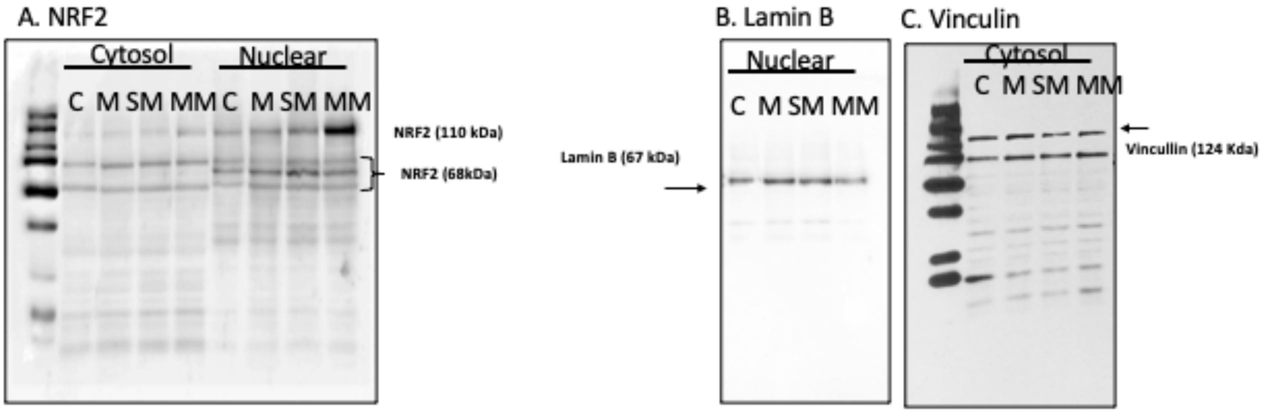

Supplemental Figure S4. Original blots for Fig. 2 E and F. MB231 cells were treated with vehicle control (C), methionine (M), seleno-methionine (SM) and MMF (MM).

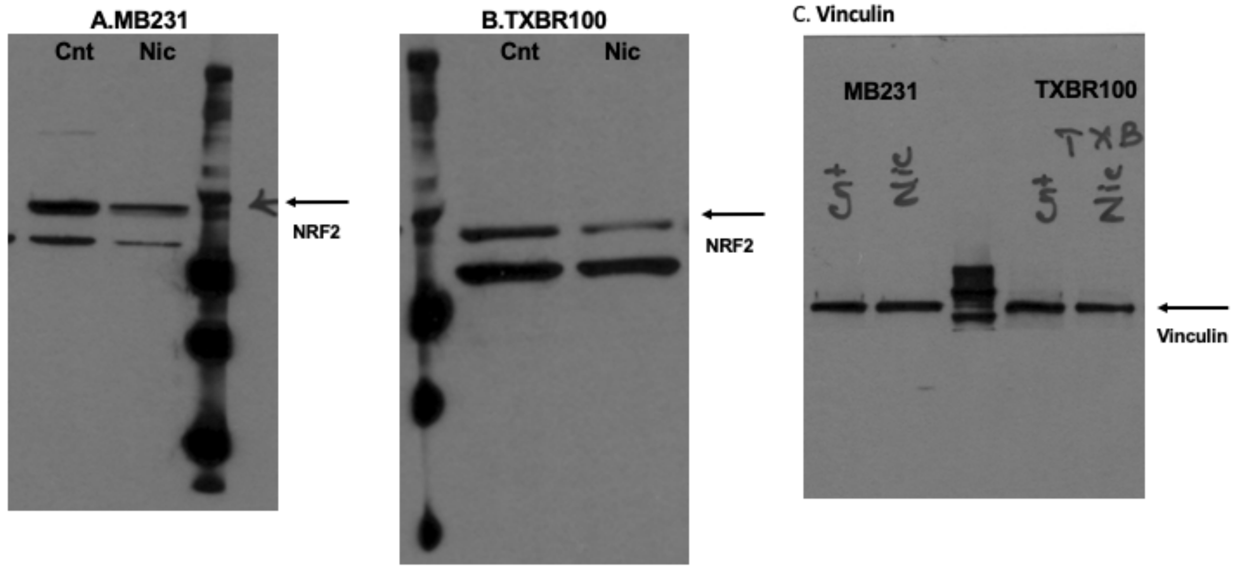

Supplemental Figure S5. Original blot for Fig. 5 A & C.

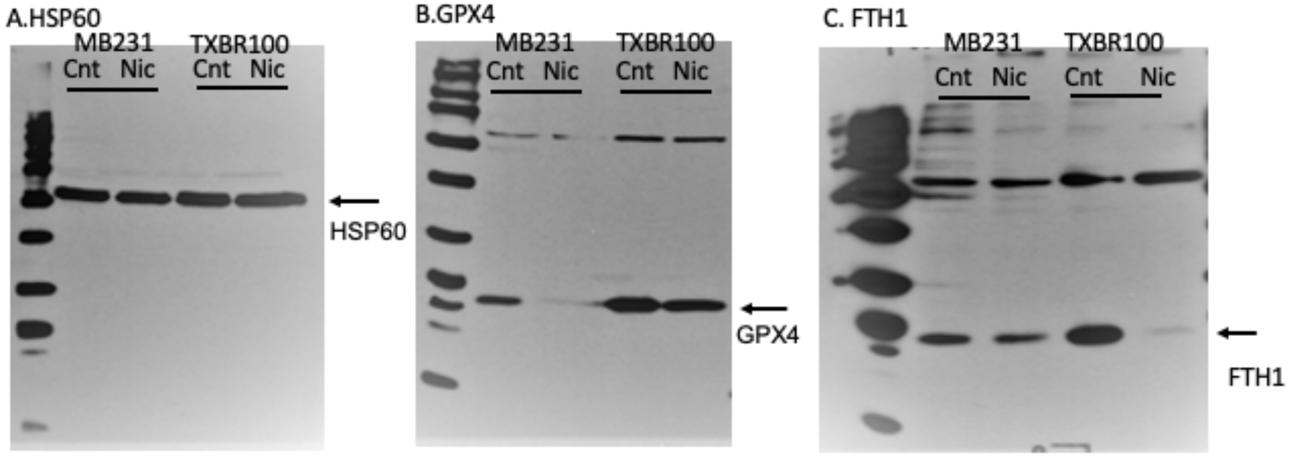

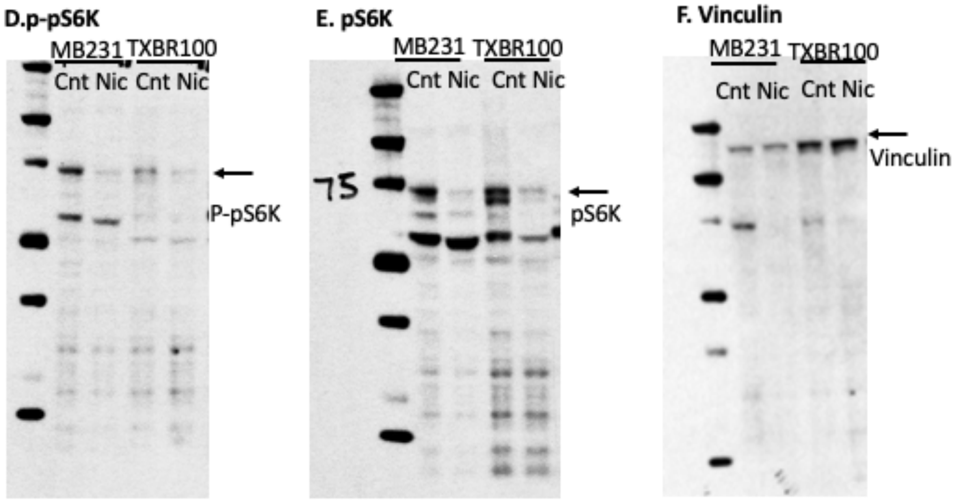

Supplemental Figure S6. Original blots for Fig. 7 E and G.

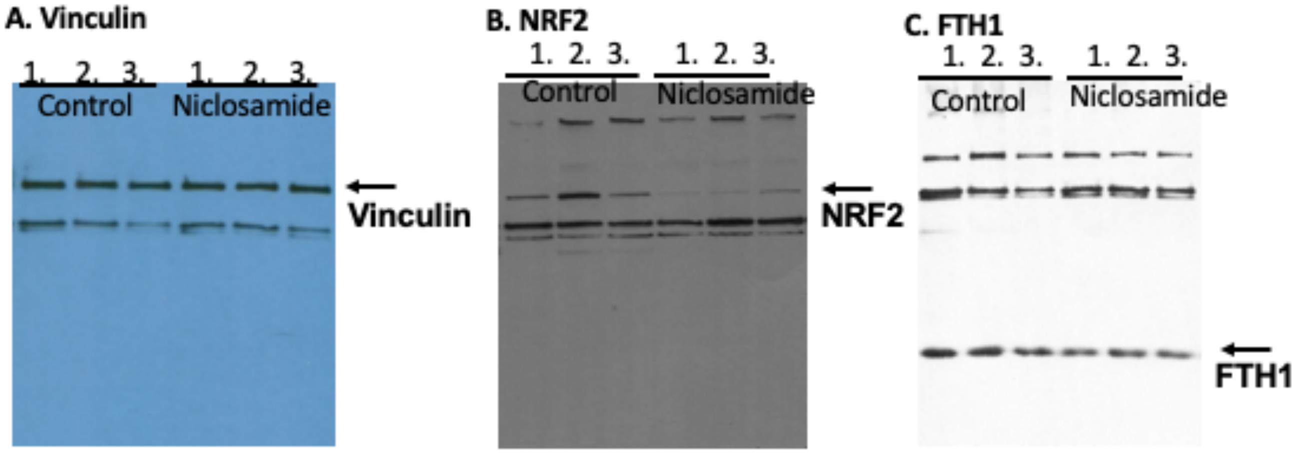

Supplemental Figure S7. Original blot for Fig. 11D.

Supplemental Table S1. Primer sequences for qRT-PCR.

Gene	Forward	Reverse
SLC38A5	GTTGGGGCCATGTCCAGT TA	AGTGTTTCATGAGGGCGA GG
SLC7A11	TGTGTGGGGTCCTGTCACTA	CAGTAGCTGCAGGGCGTATT
HO-1	ACTGCGTTCCTGCTCAACATC	GCTCTGGTCCTTGGTGTCATG
GCLM	GTGATGCCACCAGATTTGACT	CCC ACT CGT GCG CTT GAA T
GCLC	GGCACAAGGACGTTCTCAAGT	CAAAGGGTAGGATGGTTTGGG
NRF2	TCTGACTCCGGCATTTCACT	GGCACTGTCTAGCTCTTCCA
18S	CCCGTTGAACCCCATTCGT	GCCTCACTAAACCATCCAATCGGTA
